# A novel model to correlate hydrogel spacer placement, perirectal space creation, and rectum dosimetry in prostate stereotactic body radiotherapy

**DOI:** 10.1186/s13014-018-1135-6

**Published:** 2018-10-01

**Authors:** Mark E Hwang, Paul J Black, Carl D Elliston, Brian A Wolthuis, Deborah R Smith, Cheng-Chia Wu, Sven Wenske, Israel Deutsch

**Affiliations:** 1 Department of Radiation Oncology, Columbia University Medical Center, New York, New York 10032 USA; 2 Department of Urology, Columbia University Medical Center, New York, 10032 New York USA

**Keywords:** Prostate cancer, Stereotactic body radiotherapy, Rectal toxicity, SpaceOAR hydrogel, Dosimetry

## Abstract

**Background:**

The SpaceOAR hydrogel is employed to limit rectal radiation dose during prostate radiotherapy. We identified a novel parameter – the product of angle θ and hydrogel volume – to quantify hydrogel placement. This parameter predicted rectum dosimetry and acute rectal toxicity in prostate cancer patients treated with stereotactic body radiotherapy to 36.25 Gy in 5 fractions.

**Methods:**

Twenty men with low- and intermediate-risk prostate cancer underwent hydrogel placement from 2015 to 2017. Hydrogel symmetry was assessed on the CT simulation scan in 3 axial slices (midgland, 1 cm above midgland, 1 cm below midgland). Two novel parameters quantifying hydrogel placement – hydrogel volume and angle θ formed by the prostate, hydrogel, and rectum – were measured, and the normalized product of θ and hydrogel volume calculated. These were then correlated with perirectal distance, rectum maximum 1–3 cc point doses (rD_max_ 1–3 cc), and rectum volumes receiving 80–95% of the prescription dose (rV80–95%). Acute rectal toxicity was recorded per RTOG criteria.

**Results:**

In 50% of patients, hydrogel placement was symmetric bilaterally to within 1 cm of midline in all three CT simulation scan axial slices. Lateral hydrogel asymmetry < 2 cm in any one axial slice did not affect rectum dosimetry, but absence of hydrogel in the inferior axial slice resulted in a mean increase of 171 cGy in the rD_max_ 1 cc (*p* < 0.005). The perirectal distance measured at prostate midgland, midline (mean 9.1 ± 4.3 mm) correlated strongly with rV95 (R^2^ 0.6, *p* < 0.001). The mean hydrogel volume and θ were 10.3 ± 4.5 cc and 70 ± 49°, respectively. Perirectal distance, rV95 and rD_max_ 1 cc correlated with hydrogel angle θ (*p* < 0.01), and yet more strongly with the novel metric θ*hydrogel volume (p < 0.001). With a median follow up of 14 months, no rectal toxicity >grade 2 was observed. Low grade rectal toxicity was observed in a third of men and resolved within 1 month of SBRT. Men who had these symptoms had higher rD_max_ 1 cc and smaller θ*hydrogel volume measurements.

**Conclusions:**

Optimal hydrogel placement occurs at prostate midgland, midline. The novel parameter θ*hydrogel volume describes a large proportion of rectum dosimetric benefit derived from hydrogel placement, and can be used to assess the learning curve phenomenon for hydrogel placement.

## Background

Hypofractionated prostate radiotherapy has gained popularity in prostate cancer treatment, with growing evidence that showed non-inferior tumor control and similar toxicity profile when compared against conventionally-fractionated, dose-escalated radiotherapy [1–3]. Stereotactic body radiotherapy (SBRT) for low risk prostate cancer was evaluated in RTOG 0938 and offered to eligible patients as a short course of radiotherapy lasting 2.5 weeks. Following per-protocol rectum dose constraints, under one quarter of prostate SBRT patients experienced 5-point changes in one-year Expanded Prostate Index Composite bowel scores after SBRT – well under the 35% considered acceptable for patients [4]. Ongoing efforts to minimize rectum toxicity in the context of a trend toward higher doses per fraction as seen in RTOG 0938 have thus remained a priority [5].

Research has shown that perirectal space enlargement with a temporary spacer material is one approach to reduce rectum dose. In one cadaveric study, a 20 cc hydrogel spacer generated a mean perirectal distance of 12.5 mm and a four-fold decrease in calculated rectum V70 Gy [6]. Multi-institutional evaluation of a different hydrogel spacer resulted in 7.5 mm of perirectal separation and a 10% reduction in rectum V40-70Gy when comparing pre- and post-spacer treatment plans [7]. More recently, the Augmenix SpaceOAR hydrogel, which received FDA approval following a 2014 phase III clinical trial, showed statistically significant reduction in grades 1 and 2 acute rectal toxicity in men receiving conventionally fractionated, dose escalated prostate radiotherapy [8]. Late grade 1 rectal toxicity at 3 years was also significantly lower in the SpaceOAR arm (42% v 17%, *p* = 0.04) [9]. This toxicity improvement is attributable to both reduced intrafraction motion, where the SpaceOAR hydrogel and daily rectal balloon usage are believed to be comparable immobilization tools [10], and improved rectal dosimetry based on posterior, rather than anterior, displacement of the anterior rectal wall with hydrogel instead of a rectal balloon [11]. A hydrogel spacer also has the additional benefit of allowing larger planning margins and higher target coverage due to improved rectal sparing [12, 13].

Recent publications have thus far correlated hydrogel distribution with rectum dosimetry in conventionally fractionated prostate radiotherapy, and demonstrated the dosimetric superiority of hydrogel over rectal balloon in prostate SBRT [11, 14]. Yet no study has correlated the *quality* of hydrogel placement with rectum dosimetry in prostate SBRT. Optimal hydrogel placement should have a greater impact on toxicity minimization in high-dose-per-fraction SBRT than in the conventionally fractionated setting. This need to examine hydrogel placement is important in the face of research that showed patients receiving prostate SBRT doses up to 50 Gy in 10 Gy per fraction, but without use of a periprostatic spacer, suffered high grade rectal toxicity [5].

Identifying parameters that help optimize hydrogel placement will be clinically meaningful, especially in the context of rising hydrogel utilization and SBRT doses. In this study, we applied a previously published metric to analyze the symmetry of hydrogel placement in our SBRT patient cohort, developed a new metric to correlate the effect of hydrogel placement on rectum dosimetry, and report early toxicity outcomes.

## Methods

### Patients

We retrospectively reviewed all low- and intermediate-risk prostate cancer patients who were treated with linear-accelerator based SBRT to 36.25 Gy in 5 fractions. The twenty patients (Table 1) were extracted from our single-institution IRB approved databases from August 2015 to August 2017. Patients received pre- and post-hydrogel placement T2-weighted prostate MRI when possible. The post-hydrogel MRI was obtained at a median of 15 (range: 2–37) days after hydrogel placement. All patients underwent simultaneous periprostatic SpaceOAR hydrogel (Augmenix, Inc. Waltham, MA) and MRI-compatible Cybermark gold fiducial prostate marker (CIVCO Medical Instruments Co., Inc. Kalona, IA) placement 16–41 days (mean ± SD 29 ± 7.5 days) prior to starting radiotherapy. Only one SpaceOAR hydrogel kit was used per patient. All men underwent CT-based simulation, and the post-hydrogel MRI was fused to the CT simulation scan to facilitate hydrogel contouring. Normal tissue dose constraints, contours, and clinical target volume (CTV) to planning target volume (PTV) margin expansions were defined as per RTOG 0938. Prostate SBRT was planned on the Varian Eclipse treatment planning system and delivered with a Varian Truebeam Linear Accelerator (Varian Medical Systems, Palo Alto, Ca).

**Table 1:** Patient characteristics

Age ± stdev, y	69.7 ± 6.6
Range	55–82
PSA ± stdev, ng/mL	9 ± 5.3
Range	2.7–24.5
Gleason score 6 (%)	25
Gleason score 7 (%)	75
Primary GS 4 (%)	47
Cores involved ± stdev (#)	3.9 ± 2.4
Range	1–9
Cores involved ± stdev (%)	30 ± 19
Range	10–75
Clinical stage:	
cT1c	72
cT2a	17
cT2b	13
AUA score ± stdev^a^	9 ± 7
Range	0–20
SHIM score ± stdev^b^	14 ± 8.7
Range	0-26
Prostate volume ± stdev (cc)	40.1 ± 22.5
Range	13–108

^a^AUA = American Urological Association Symptom Score

^b^SHIM = Sexual Health Inventory for Men Score

### Quantifying SpaceOAR hydrogel placement

SpaceOAR hydrogel symmetry was first analyzed according to the rubric outlined by Fischer-Valuck et al. in patients receiving conventionally fractionated radiotherapy [14]. In their study, three axial slices on the CT simulation scan were selected for analysis (midgland, 1 cm above migland, and 1 cm below midgland). The presence or absence of hydrogel in each slice was recorded. Hydrogel placement was recorded as asymmetric if the hydrogel was present but deviated by greater than 1 cm from anatomic midline. Each patient received a composite hydrogel symmetry score that was compared with their final rectal dosimetry: Symmetry Score 1 (SYM1) = all 3 slices with symmetric distribution; Symmetry Score 2 (SYM2) = 1 of 3 slices with > 1 cm but < 2 cm asymmetry; Symmetry Scores 3–5 (SYM 3–5) were defined in their original publication as having progressively prominent asymmetry, but which were not needed in our study.

The SpaceOAR hydrogel, rectum, and CTV were identified and contoured on each patient’s CT simulation scan. A post-hydrogel MRI T2-weighted sequence was fused with the CT simulation scan to facilitate hydrogel delineation. Volumes and center-of-mass coordinates were recorded for the prostate CTV, rectum, and hydrogel.

**Fig. 1:**
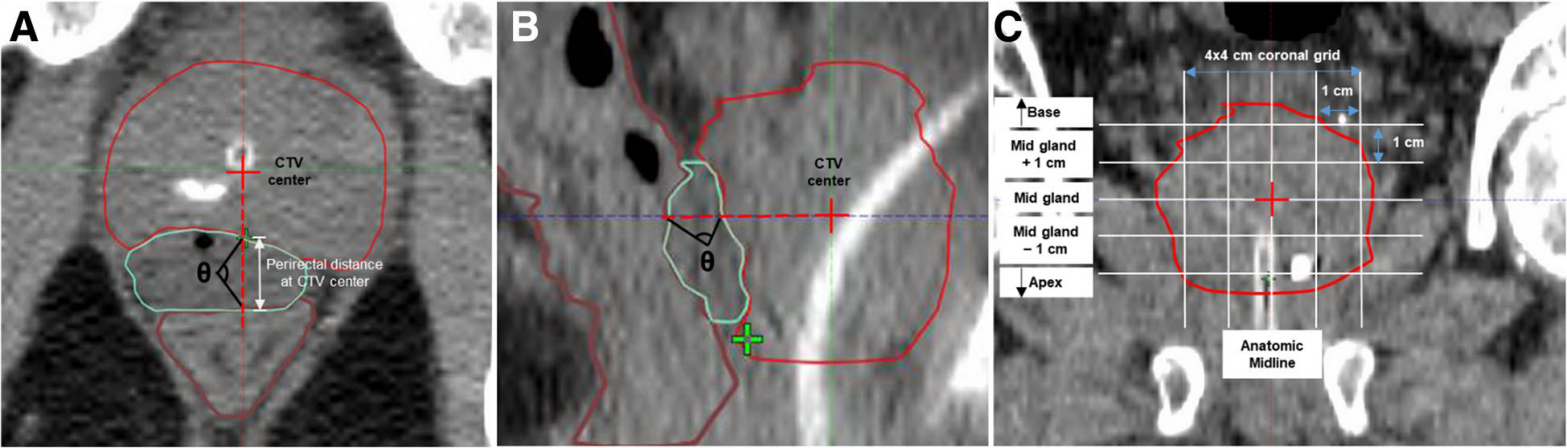
Measurement of a perirectal distance at CTV center on axial CT image (**a**), and θ in 3-dimensional space on axial and sagittal CT images (**a**, **b**). The perirectal distance is measured at each vertex in a 4 × 4 cm coronal grid centered on the prostate midgland, at midline to generate a perirectal space map comprising up to 25 perirectal distance measurements for each patient (**c**)

We quantified the hydrogel deviation from midline by measuring the angle, θ, formed by the posterior aspect of the CTV at CTV center (corresponding to prostate midgland, midline), hydrogel center of mass, and anterior wall of the rectum in 3-dimensional space (Fig. 1a, b).

Patient rectal toxicities were recorded per RTOG grading during treatment, 2–4 weeks after treatment, and every 3 months thereafter [15].

### Quantifying the perirectal space

We measured the distances between the prostate and rectum at different locations to create a perirectal space map for each patient before and after hydrogel placement. Each perirectal distance was bounded anteriorly by the posterior aspect of the CTV, and posteriorly by the anterior portion of the rectum contour. We first measured the perirectal distance at the CTV center, which corresponded to the prostate midgland, at midline (Fig. 1a). Additional perirectal distances were then measured up to 2 cm superiorly, inferiorly, and bilaterally from the CTV center in 1 cm intervals. The perirectal space for each patient is thus mapped with 25 potential perirectal distance measurements that are spatially arranged in a 4 × 4 cm coronal grid (Fig. 1c). These measurements were performed on the CT simulation scan (*n* = 20), and repeated on each patient’s pre- (*n* = 13) and post- (*n* = 18) hydrogel placement MRI T2-weighted scans. The *change* (Δ, n = 13) in perirectal space attributed to hydrogel placement was determined by subtracting the post- from pre-hydrogel perirectal distance measurements on MRI when both MRI scans were available. Perirectal distance measurements were then compared with the volume of rectum receiving 95, 90, and 80% of the prescription dose (rV95–80), as well as rectum maximum 1, 2, and 3 cc point doses (rD_max_ 1–3 cc).

### Statistical analysis

Statistical significance between SYM1 and all other symmetry groups was evaluated with student *t* test. Continuous variables were summarized with means and standard deviations and shown to approximate a normal distribution with a Shapiro-Wilk normality test. The relationships amongst continuous variables were quantified using Pearson correlation and multiple regression analysis. Analysis was performed in Office Suite Excel 2013 (Microsoft Corporation, Redmond, WA) and SPSS (IBM, Armonk, NY).

## Results

### SpaceOAR symmetry analysis

Hydrogel symmetry was analyzed on three axial slices from each patient’s CT simulation scan, for a total of 60 analyzed axial slices from our cohort of 20 patients. SpaceOAR hydrogel was present in 51 axial slices and symmetric to within 1 cm of anatomic midline in 45 axial slices. Lateral hydrogel asymmetry was present in six slices but did not exceed 2 cm from midline. Hydrogel was completely absent in nine axial slices: four inferior to midgland, two at midgland, and three superior to midgland.

Thirteen patients (65%) had hydrogel present in all three axial slices. Ten patients (50%) had hydrogel present and symmetric bilaterally in all three axial slices, while three pateints (15%) had hydrogel present in all three axial slices but symmetric in only two of them (SYM 2). None of our patients met criteria for categorizing hydrogel placement into scores SYM3–5 (that denote asymmetry > 2 cm or asymmetry > 1 cm in more than one of three axial slices). SYM scores were not assigned to the remaining seven patients, five of whom had hydrogel in only two axial slices and two of whom had hydrogel in only one axial slice.

**Fig. 2:**
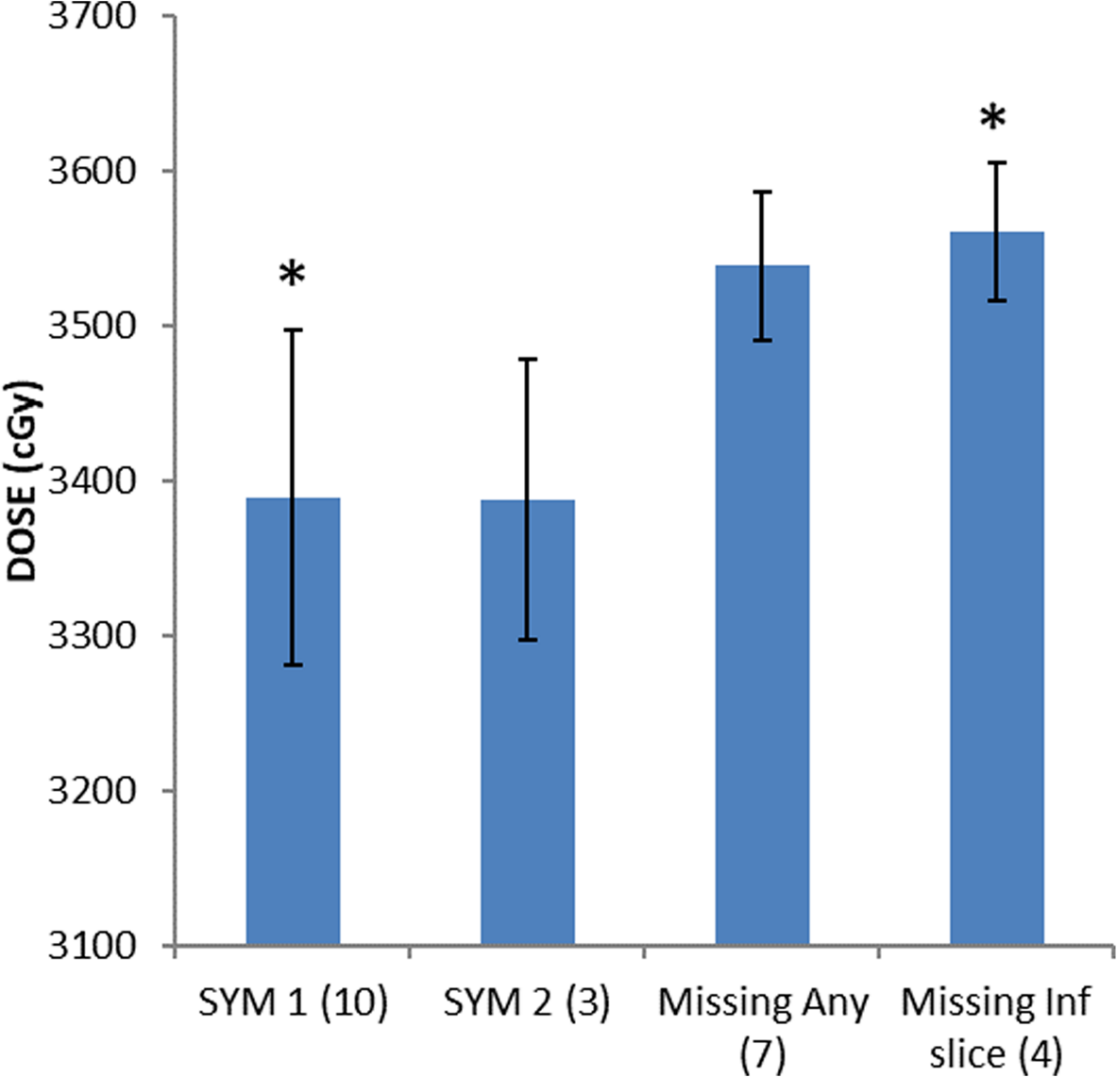
Rectum maximum 1 cc point dose (rD_max_ 1 cc) by hydrogel symmetry (SYM) score. T-test comparison of means revealed a statistically significant difference between groups SYM1 and Missing Inf Slice, denoted by * = *p* < 0.01

The rD_max_ 1 cc dose was not statistically different between groups SYM1 and SYM2 (Fig. 2). This is similar to results shown by Fischer-Valuck et al. [14]. However, rD_max_ 1 cc was on average 171 cGy higher in patients missing hydrogel in the inferior most axial slice versus patients with SYM1 scores (Fig. 2, *p* < 0.005).

### Perirectal distance analysis on post-hydrogel simulation CT

Perirectal distance measurements from patient CT simulation scans are shown in Table 2. The mean perirectal distance measured at CTV center was 9.1 ± 4.3 mm. Perirectal distances were greatest at the superior-most axial slice, averaging 14.7 ± 10.3 mm, and smallest at the inferior-most slice, averaging 6.4 ± 4.5 mm. Averaging all perirectal distance measurements for each patient, the mean perirectal distance was 10.1 ± 7.4 mm, which closely approximated the single perirectal distance obtained at CTV center for each patient.

**Table 2:** Mean perirectal distances (mm) measured on the CT simulation scan (*n* = 20). Standard deviation immediately beneath corresponding perirectal distance. The mean perirectal distance and standard deviation at each of 5 axial slices (right) and 5 sagittal slices (bottom) are also reported

		Left		Midline	Right		Mean	Stdev
		2 cm	1 cm	0	1 cm	2 cm		
Superior (base)	+ 2 cm	14.4	12.5	14.5	13.8	18.1	14.7	*10.3*
		*6*	*8.2*	*11.1*	*9.6*	*16.9*		
	+ 1 cm	19.1	11	9.9	11.3	13	12.9	*7.5*
		*11.2*	*7.5*	*5.8*	*5.9*	*10.7*		
Midgland	0	11.9	9	9.1	10.5	11.9	10.5	*5.3*
		*4*	*5.3*	*4.3*	*5.5*	*12.2*		
Inferior (apex)	−1 cm	8.7	8.2	8.5	8.2	9.4	8.6	*5.6*
		*2.6*	*5.9*	*5.1*	*6.8*	*2.2*		
	−2 cm		7.6	5.5	6.1		6.4	*4.5*
			*4.3*	*4.2*	*4.4*			
	Mean	13.5	9.7	9.5	10	13.1	10.1	*7.4*
	Stdev	*7.7*	*6.4*	*6.9*	*6.9*	*12.5*		

In none of our cohort was perirectal distance measurement possible in the inferior-most slice, 2 cm to the left or right of midline, due to an absence of either CTV or rectum contour at this location.

### Perirectal distance analysis on pre- and post-hydrogel MRI

Perirectal distances measured on the post-hydrogel MRI T2 sequence (Table 3B) were compared with those measured on post-hydrogel CT simulation scan. The perirectal distance at any given perirectal location was not statistically significantly different on t-test comparison of means between the post-hydrogel CT and MRI in the 18 men who had both scans (not shown, data available upon request). The greatest increase (Δ) in perirectal distance after hydrogel placement occurred at midline on the midgland axial slice (CTV center), and averaged 10 mm (Table 3C). The Δ perirectal space diminished at distances greater than 1 cm from CTV center.

**Table 3:** Mean perirectal distances (mm) on the T2-weighted MRI before (A, n = 13), and after SpaceOAR hydrogel (B, n = 18 patients). Mean change (Δ) in perirectal distances (C, *n* = 13)

			Left		Midline	Right	
			2 cm	1 cm	0	1 cm	2 cm
A	Superior (base)	+ 2 cm	12	9.1	11.7	10	14.3
		+ 1 cm	12.8	8.2	5.8	6.8	10.8
	Midgland	0	8.3	3	1	2.5	6.5
	Inferior (apex)	−1 cm	6.5	1.8	0.6	2	7.6
		−2 cm		3	1.5	5	
B	Superior (base)	+ 2 cm	15	13.6	14.6	12.2	16.2
		+ 1 cm	13.7	11.8	10.9	10.9	12
	Midgland	0	7.4	9.7	10.3	9.6	7
	Inferior (apex)	−1 cm	7	8.7	10.3	9.2	10.6
		−2 cm		8.1	7.3	6.8	
C	Superior (base)	+ 2 cm	0.5	3.5	4.8	4.6	2
		+ 1 cm	0	3.6	5.5	5.3	1
	Midgland	0	0	7.6	10	8.1	2.3
	Inferior (apex)	−1 cm	0	7.9	9.8	8.8	1.3
		−2 cm		5.2	5.9	4.5	

### Rectum dosimetry

RTOG 0938 rectum dose constraints were met in 18 of 20 patients. These constraints limited rD_max_ 1, 2 and 3 cc point doses, and rV95, rV90 and rV80. Two patients received rD_max_ 3 cc rectum doses of 3448 and 3460 cGy, which was slightly in excess of the 3440 cGy constraint.

Table 4 shows R^2^ values from regressing different measures of rectum dosimetry against independent variables - perirectal distances, hydrogel volume, θ, and θ*hydrogel volume. The perirectal distance that explained the highest rectum dosimetry variance was that measured at CTV center (Table 4A, first row). Perirectal distance measurements that were inferior to this location, representing the perirectal space that is natively most closely approximated to the rectum, have slightly lower ability to explain rectum dosimetry variance (Table 4A, rows 2–3). Variance explanation strength decreased precipitously with perirectal distance measurements obtained further from the CTV center in all directions (results not shown).

**Table 4:** Coefficient of determination (R^2^) values from simple linear regression models of rectum maximum 1, 2, and 3 cc point doses (rD_max_ 1, 2, 3 cc) and rectum volume receiving dose (rV95, 90, 80%) regressed against perirectal distance measurements obtained on CT (A) and hydrogel parameters (B)

		rD_max_			rV		
		1 cc	2 cc	3 cc	95%	90%	80%
A	Perirectal Distance Measurement						
	CTV center	0.44	0.45	0.42	0.6	0.55	0.27
	1 cm inferomedial to CTV center	0.41	0.37	0.31	0.5	0.38	0.15
	2 cm inferomedial to CTV center	0.39	0.35	0.28	0.49	0.33	0.1
B	Hydrogel Parameters						
	Hydrogel volume (cc)	0.23	0.22	0.18	0.2	0.14	0.04
	θ	0.36	0.33	0.26	0.43	0.34	0.14
	Normalized θ*gel volume	0.64	0.62	0.55	0.60	0.52	0.25

Mean contoured hydrogel volume was 10.3 ± 4.5 cc, and mean θ was 70 ± 49°. Hydrogel volume and θ individually showed moderate power in explaining rectum dosimetry variance (Table 4B), with R^2^ < 0.43 for any of rV80–95 and rD_max_ 1–3 cc. The product of θ, normalized to 180°, and hydrogel volume, normalized to the volume of the single largest contoured hydrogel in this cohort, showed high power in explaining rectum dosimetry (R^2^ for rV95 = 0.60, *p* < 0.001; rD_max_ 1 cc = 0.64, p < 0.001). CTV volume, with a mean and standard deviation of 40.1 ± 22.5 cc, had minimum ability to explain rectum dosimetry variance (not shown).

**Fig. 3:**
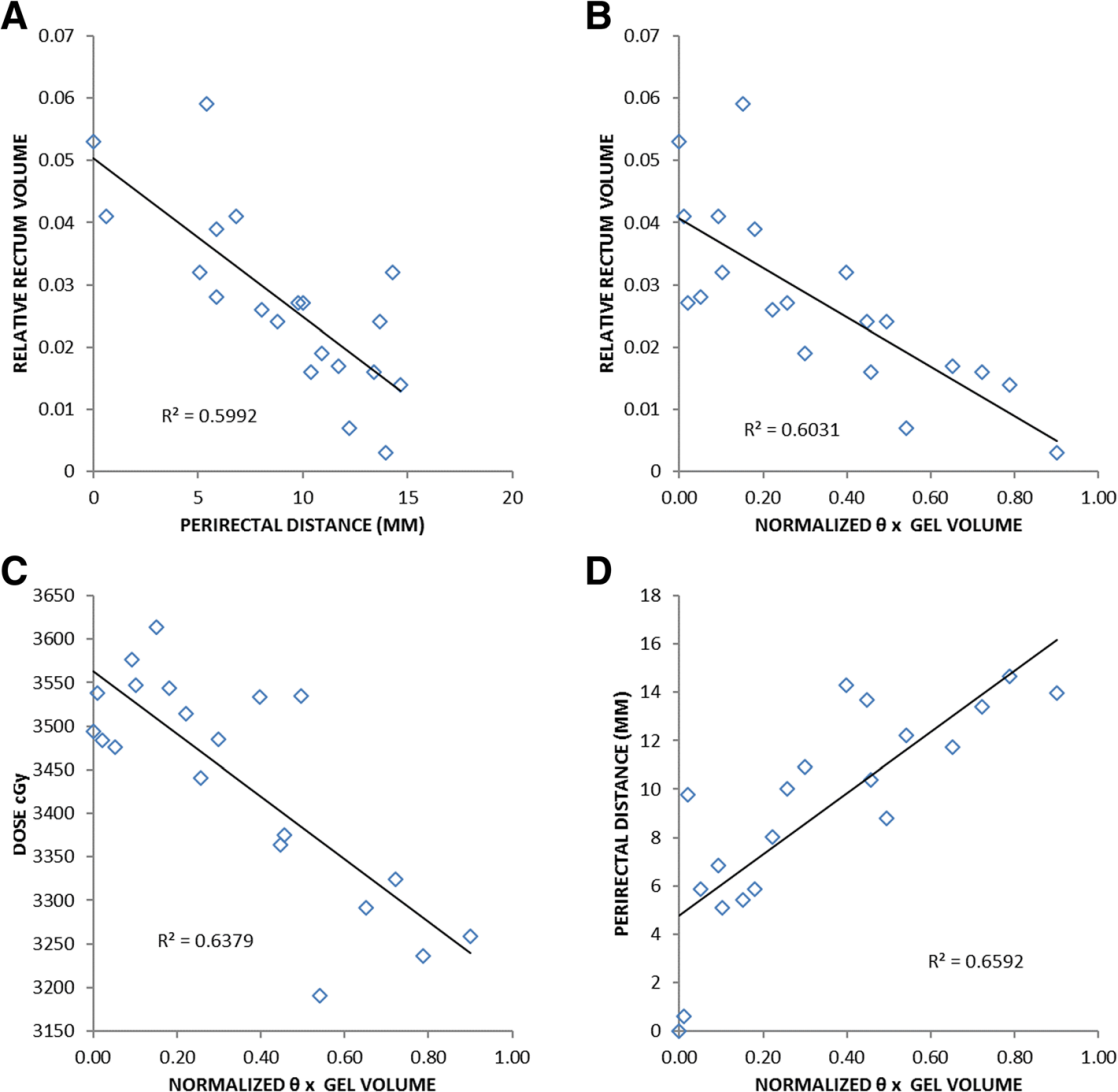
Perirectal distance at CTV center versus rV95%, R^2^ 0.60, p < 0.01, (**a**). Normalized θ*hydrogel volume versus: rV95%, R^2^ 0.60, p < 0.01, (**b**); rD_max_ 1 cc, R^2^ 0.64, p < 0.01, (**c**); and perirectal distance at CTV center, R^2^ 0.66, p < 0.01, (**d**)

The parameter, θ*hydrogel volume, was as strongly correlated with, and explained the same proportion of variance in, rV95 as did the single perirectal distance measurement taken at CTV center (R^2^ 0.6, Table 4A, B; Fig. 3a, b). θ*hydrogel volume was even more strongly correlated with rD_max_ 1, 2 and 3 cc point doses than with any single perirectal distance measurement (Table 4A, B; Fig. 3c). This suggests that θ*hydrogel volume successfully describes the perirectal space creation effect of a given hydrogel (Fig. 3d).

Multiple linear regression was then used to identify predictive effects of independent variables θ, hydrogel volume, and θ*hydrogel volume on dependent variable rD_max_ 1 cc. Only the independent variable θ*hydrogel volume was a significant predictor of rD_max_ 1 cc, indicating a significant interaction component between θ and hydrogel volume in predicting rectum dosimetry (Standardized β = − 1.62; *t* > − 3.05; R^2^ = 0.694; F = 12.08 (19,3)).

### Acute rectal toxicity

**Fig. 4:**
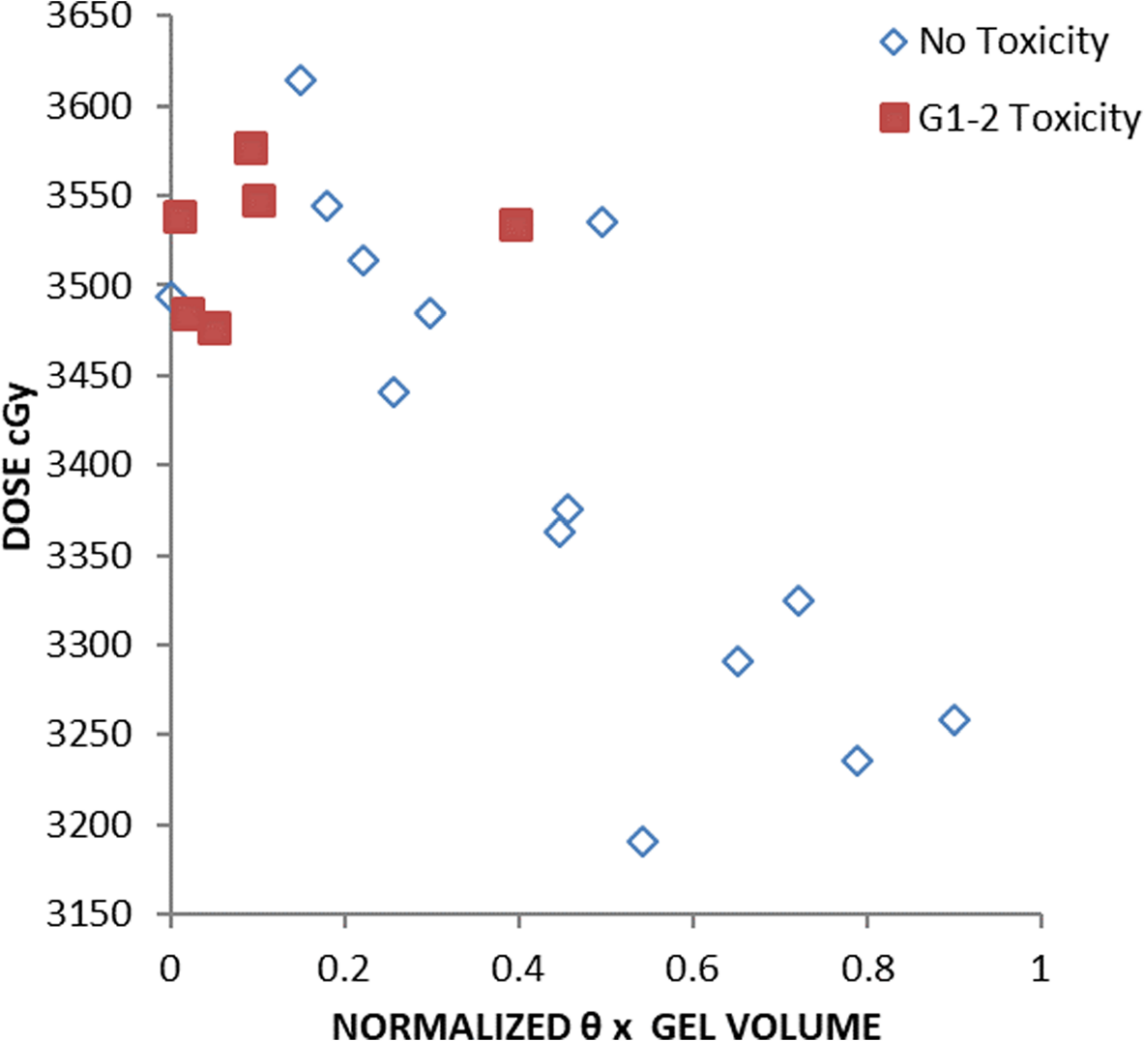
Normalized θ*hydrogel volume versus rectum maximum 1 cc point dose (rD_max_ 1 cc), showing acute low grade rectal toxicity (*n* = 6/20)

With a median follow up of 18 months, 30% of men experienced Grade 1 (*n* = 5) or 2 (*n* = 1) acute rectal toxicity with soft stools during treatment. Symptoms completely resolved within two weeks in four men, and within four weeks in the remaining two men. No GI toxicity was reported on any subsequent follow up. Men with acute rectal toxicity tended to have higher rD_max_ 1 cc and lower θ*hydrogel volume scores (Fig. 4). No toxicity greater than Grade 2 was observed.

## Discussion

### Clinical relevance

Our institutional experience describes the early stages of SpaceOAR hydrogel implementation in high-dose per fraction SBRT setting, where the benefits of a well-placed spacer on rectum dosimetry quickly become clinically evident upon examination. Our objective was to develop a systematic method to quantify the perirectal space after hydrogel placement and identify a hydrogel placement metric to correlate perirectal space creation with rectum dosimetry. The findings showed increased perirectal space and optimal hydrogel placement have a positive impact on rectal dosimetry when we considered the θ*hydrogel volume metric. This metric was at least, if not more, predictive of rectum dosimetry than any perirectal space measurement. With longer follow up and greater sample size, this metric should have added utility of eventually tracking placement quality and operator experience.

Evidence of a learning curve in developing operative skillsets is well-established as seen in prostate brachytherapy implant quality that improves with experience up to a point [16, 17], after which proficiency is maintained with a minimum annual caseload [18]. The learning curve phenomenon for hydrogel placement was first reported by Pinkawa et al. in a study of 64 patients that showed improved lateral hydrogel symmetry, increased perirectal space, and better rectum dosimetry in the latter 32 patients compared with the first 32 patients [19]. Such a learning-curve effect is minimized in a well-established training environment with appropriate mentorship and operator feedback [20]. We expect that a well-designed hydrogel placement metric, such as we have described in this manuscript, would provide one such measure of feedback.

### Quantifying perirectal space

While results from this study are in agreement with the general expectation that a well-placed hydrogel is important for rectum dosimetry, to our knowledge this is the first time the post-hydrogel perirectal space has been rigorously mapped. We showed that precise perirectal space measurements can be obtained independently from either the post-hydrogel T2 weighted MRI or CT simulation scans (Tables 2 and 3). Prior to hydrogel placement, the prostate apex and midgland lie close to the rectum on the pre hydrogel placement MRI (Table 3A). Following hydrogel placement, almost all regions of the prostate have increased separation from the rectum, with the greatest mean increase seen at the prostate apex and midgland (Table 3C). Several publications have described a wide range of post-hydrogel perirectal separation ranging from 0.6 to over 2 cm, but to-date few have described in detail the optimal location to obtain this perirectal measurement, or Δ measurement, resulting from hydrogel placement [11, 14, 21–23].

From our perirectal space maps, we identified the perirectal distance measurement obtained posterior to the prostate at midgland, midline, or CTV center, as that which is most strongly correlated with rectum dosimetry (Table 4A). This is followed by the perirectal distance measurements 1 and 2 cm that are immediately inferior to it i.e. toward the prostate apex. As enlargement of the perirectal space at, or slightly inferior to, the prostate CTV center led to the greatest improvement in rectum dosimetry, this position represented the optimal location for hydrogel placement.

### SpaceOAR characteristics and rectum dosimetry

Distribution of lateral hydrogel deviation in our patient cohort was nearly identical to that of earlier work by Fischer-Valuck et al. [14] Half (*n* = 10) of patients had symmetric gel placement (SYM1, lateral deviation < 1 cm) in all three axial slices, and 15% (*n* = 3) had hydrogel in all three axial slices but with lateral deviation < 2 cm in only one axial slice (SYM2). Consistent with their conclusions, the rV95% and rD_max_ 1 cc were not significantly different between SYM1 and SYM2 hydrogels in our study (Fig. 2).

While the effect of lateral hydrogel deviation on rectum dosimetry is thus well-characterized, hydrogel distribution along the craniocaudal dimension has not been commented upon in existing literature. In our study minor hydrogel deviation along the craniocaudal axis had a more pronounced effect on rectum dosimetry than lateral deviation of a similar scale (~ 1 cm). The absence of hydrogel in the axial slice 1 cm inferior to midgland (*n* = 4) correlated to an increase in rD_max_ 1 cc of 171 cGy (*p* = 0.005, Fig. 2).

This is unsurprising as the native perirectal space is most limited inferomedially, averaging 1–2 mm in our analysis. In the vast majority of patients, such close proximity between the prostate and rectum begins inferiorly at the prostate apex and extends superiorly at least as far as the prostate midgland. Whereas previous analysis emphasized the importance of lateral hydrogel symmetry, we conclude that hydrogel deviation must be accounted for in both the lateral and cranio-caudal dimensions to accurately predict rectum dosimetry.

As a result, we defined θ to quantify hydrogel deviation from the optimal CTV center location in three-dimensional space rather than along a single axis. θ alone correlated moderately with rD_max_ 1 cc and rV95% and predicted only a minority of variance in these statistics (R^2^ = 0.36 and 0.43, respectively; Table 4B) as it does not take into account the volume of hydrogel centered around the θ vertex.

Similarly, hydrogel volume alone was modestly correlated with rectum dosimetry (R^2^ rD_max_ 1 cc = 0.23; rV95%, 0.2). This is due to minimal enlargement of the perirectal space in the instance of suboptimal injection site (i.e. small θ). Indeed, hydrogel volume was not significant in two-variable regression of hydrogel volume and θ against rD_max_ 1 cc (p_VOLUME_ = 0.06, p_θ_ < 0.01, *n* = 20), but became significant only in the subset of patients with θ > 35° (p_VOLUME_ = 0.04, p_θ_ < 0.01, *n* = 16).

We thus inferred that an interaction exists between hydrogel volume and θ such that the correlation of each θ and hydrogel volume influenced rectum dosimetry. Based on this examination of data, we tested the product θ*hydrogel volume which showed strong ability to explain rectum dosimetry (R^2^ rD_max_ 1 cc =0.64; rV95%, 0.6; Table 4B). A three-variable regression analysis of θ, hydrogel volume, and θ*hydrogel confirmed that θ*hydrogel was the only significant predictor of rV95 (R^2^ **=** 0.694; F**-**value 12.08; *p* = 0.008). We thus conclude that the parameter θ*hydrogel volume quantifies perirectal space enlargement effect following hydrogel placement, and should be considered in evaluating hydrogel placement success.

Previous work has shown no statistically significant variation in rectum dose with CTV volume ranging up to 100 cc, and is consistent with our findings [21].

### Acute rectal toxicity and θ*hydrogel volume

Hydrogel spacer use in prostate SBRT was documented as early as 2013 by Alongi et al. and continues to represent a growing proportion of SpaceOAR utilization [11, 24, 25]. Yet as of this study, the only published phase III randomized prostate hydrogel data were obtained in the conventionally fractionated setting [22]. In their control arm without hydrogel, acute rectal toxicity ≥grade 2 benefit was not statistically significant, but any late toxicity was improved 4.5-fold at 3 years.

We expected that relative toxicity benefits attributed to hydrogel use would be at least as prominent in the high-dose per fraction SBRT setting as it is in conventionally-fractionated treatment. In addition, projected cost-benefit decision analysis suggests that the use of hydrogel spacer will be cost-effective for toxicity management in the long term for all forms of prostate radiotherapy, but particularly so for prostate SBRT [26].

With the hydrogel spacer and prostate SBRT to a dose of 3625 cGy in five twice-weekly treatments, less than one third of patients developed grades 1–2 acute rectal toxicity either during or immediately after treatment, which resolved at the latest by the one-month post-treatment visit. It is noteworthy that these men had characteristics of rectum dosimetry, perirectal spacing, and θ*volume that we quantified as being in the less favorable half of our cohort (Fig. 4).

Likely as a result of small sample size, no statistically significant difference was observed in rD_max_ 1 cc, rV95%, perirectal distance at CTV center, and the metric θ*hydrogel volume, between men who developed acute rectal toxicity and men who did not. We suspect that the likelihood of statistically significant relationships between these parameters and symptomatic toxicity would emerge at the higher SBRT doses currently being evaluated in phase II clinical trials, with larger sample size, and longer follow-up.

We have nonetheless shown from our early hydrogel SBRT patient cohort a correlation between the metric θ*hydrogel volume and rectum dosimetry. We expect that the learning curve necessary to attain high-quality hydrogel placements will also become evident as measured by the θ*hydrogel volume metric as our experience with hydrogel placement increases.

The relatively wide range of hydrogel volumes as measured on post-hydrogel T2 weighted MRI bears further evaluation. Each SpaceOAR hydrogel is injected transperineally as a 10 cc suspension following dissection of Denonvillier’s fascia with saline solution. Potential explanations for small hydrogel volumes include suboptimal placement, hydrogel dispersion prior to polymerization and incidental withdrawal of hydrogel along with the needle following the procedure. Explanations for large hydrogel volumes include contouring software volume overinterpolation and overcontouring of hydrogel. The latter may occur as saline solution used in the fascial dissection and SpaceOAR hydrogel is indistinguishable on the MRI T2 image obtained after hydrogel placement. In our cohort, the earliest post-hydrogel MRI was obtained two days after hydrogel placement.

## Conclusions

After SpaceOAR hydrogel placement, the perirectal distance measurement at CTV center, which corresponds to the prostate midgland, midline, is shown to be highly correlated with rectum dosimetry. Hydrogel location can be quantified by θ as measured from the CTV center, with larger θ correlating with better placement. Hydrogel volume contributed more to perirectal space creation with increasing θ. The overall rectum dosimetric benefit from hydrogel placement strongly correlated with the novel metric θ*hydrogel volume, which can be used to track and minimize the learning curve phenomenon for hydrogel placement.

## Abbreviations

CTV: clinical target volume
PTV: planning target volume
SBRT: stereotactic body radiotherapy
SYM: Symmetry Score
